# Evaluation of *Clostridium autoethanogenum* protein as a new protein source for broiler chickens in replacement of soybean meal

**DOI:** 10.5713/ab.23.0419

**Published:** 2024-04-01

**Authors:** Xing Chen, Aijuan Zheng, Ahmed Pirzado Shoaib, Zhimin Chen, Kai Qiu, Zedong Wang, Wenhuan Chang, Huiyi Cai, Guohua Liu

**Affiliations:** 1 Key Laboratory for Feed Biotechnology of the Ministry of Agriculture and Rural Affairs, Institute of Feed Research, Chinese Academy of Agriculture Sciences, Beijing 100081, China

**Keywords:** Antioxidant Capacity, Broiler Chickens, *Clostridium autoethanogenum* Protein, Growth Performance, Immunoglobulin

## Abstract

**Objective:**

The object of this study was to investigate the effect of replacing soybean meal with *Clostridium autoethanogenum* protein (CAP) in broiler diets on growth performance, blood indicators, antioxidant capacity, and immune function.

**Methods:**

A total of 180 Arbor Acres broilers were randomly divided into three treatments, each treatment with six replicates and 10 broilers per replicate for a 42-day feeding trial. The control group (CON) was fed corn-soybean meal based diet. The CAP-1 and CAP-2 groups were considered to use CAP to replace 25% or 50% of soybean meal in the diet, respectively. The average daily gain and average daily feed intake of broilers at 1 to 21 d, 22 to 42 d, and 1 to 42 d were measured, and the feed conversion ratio was calculated. At the 42nd day of age, two broilers with similar weights and fasted for 12 h were selected in each replicate for blood collection from the brachial wing vein. The blood routine indicators, serum biochemical indicators, serum antioxidant capacity, and immunoglobulin content of broiler chickens were measured.

**Results:**

Replacement of soybean meal with 25% (CAP-1) and 50% (CAP-2) CAP significantly increased the average daily gain of 22 to 42 d and 1 to 42 d and decreased the average daily feed intake and feed conversion rate (p<0.05). The CAP-1 group, and CAP-2 group significantly increased hemoglobulin in the blood of broilers, while the CAP-2 group increased hematocrit content (p<0.05). Compared with the control group, the contents of superoxide dismutase and immunoglobulin A in serum of the CAP-2 group were significantly increased, while the contents of malondialdehyde in CAP group were significantly decreased (p<0.05).

**Conclusion:**

Replacing soybean meal with CAP led to significant improvements in the growth performance, antioxidant capacity, and immunoglobulin content of broilers.

## INTRODUCTION

Proteins play a crucial role as the fundamental components of body tissues and contribute significantly to the growth and upkeep of poultry muscles [1,2]. In the commercial poultry industry, over 70% of the production costs are attributed to feed, with soybean meal (SBM) the main feed for the broiler chickens, its price is a key factor for the cost [3]. Due to the increasing costs and source restricting of SBM, alternative protein sources should be urgently developed and utilized in feed production to reduce the industry’s reliance on SBM [4,5].

Single cell protein (SCP), also referred to microbial protein, is a protein-rich substance that contains amino acids, vitamins, minerals, nucleotides, and immune polysaccharides [6,7]. *Clostridium autoethanogenum* protein (CAP) is a newly developed and efficient SCP [8]. It is produced as a byproduct during the ethanol production process by utilizing carbon monoxide and ammonia from steelmaking waste gas [9]. The CAP is obtained through centrifugation and drying separation of the fermentation broth produced the *Clostridium autoethanogenum*, which has high protein content and a well-balanced amino acid profile [10]. Currently, no virulence genes have been found in the genome sequence of *Clostridium autoethanogenum*, and CAP contains fewer antinutritional factors when compared to traditional plant and animal protein sources [9,11]. In aquaculture, replacing fish meal in feed with CAP has a positive impact on the growth and development of aquatic animals such as herring, grass carp, and carp, which has confirmed its safety and feasibility in feed [12].

Only one previous has reported that replacing 29% SBM with CAP in the diet significantly improved the production performance and antioxidant capacity and increased the abundance of beneficial bacteria of Cobb broilers in the gut [7]. However, variations in the level of CAP addition and the use of different poultry breeds may lead to differences in outcomes. In the initial phase of this experiment, there was a systematic analysis of the chemical and amino acid composition of CAP, along with the evaluation of protein and amino acid digestibility, metabolic energy value, and net energy value of CAP in the ileum of Arbor Acres broiler chickens. Based on the above analysis, this study investigated the impact of CAP on the growth performance, blood parameters, antioxidant capacity, and immune function of Arbor Acres broilers. The findings offer valuable insights into the potential application of CAP in broiler chickens.

## MATERIALS AND METHODS

### Ethics statement

The research was licensed by the ethical approval of the Animal Care and Use Committee of the Institute of Feed Research of the Chinese Academy of Agricultural Science (Statement No. AEC-CAAS-20191106, Beijing, China). The CAP was kindly provided by Xinjiang Jinlan Plant Protein Co., Ltd. (Xinjiang, China). The chemical composition, ileal protein, and amino acid digestibility of CAP were analyzed, and the results were presented in Table 1 (chemical composition), Table 2 (ileal protein digestibility), and Table 3 (amino acid digestibility). The analysis methods of chemical composition, ileal protein, and amino acid digestibility were referenced on articles published in our laboratory [13].

### Animals and diets

A total of 180 Arbor Acres broilers with the same genetic background and similar body weight (48.28±0.05 g) aged 1-day-old were arranged in metal cages (140 cm×70 cm×40 cm, Xiping Mufeng agricultural and animal husbandry equipment Co., Ltd., Henan, China). The broilers were randomly divided into three treatments, each treatment with six replicates and 10 broilers per replicate for a 42-day feeding trial. The control group (CON) was fed corn-SBM based diet, which was formulated to meet or exceed the nutrient requirements of broilers [14]. The CAP-1 group used CAP to replace 25% of SBM, while the CAP-2 group replaced 50% of SBM in the diet. The ingredients and nutrient content of the experimental diets are shown in Table 4, ensuring the same digestive energy and crude protein for each processing group. The Arbor Acres broilers were raised in Nankou Experimental Base of Chinese Academy of Agricultural Sciences (Changping, Beijing, China). Broilers were raised in environmentally controlled facilities with fiberglass feeders and plastic grid floors, with feed and purified water provided *ad libitum*. Adjust test conditions such as temperature, humidity, and light to meet Arbor Acre broiler management guidelines. Newcastle disease virus vaccine was inoculated on the 7th and 21st day and inactivated infectious bursal disease vaccine was inoculated on the 14th and 28th day (Shanghai Haili Biotechnology Co., Ltd., Shanghai, China).

### Performance parameters

Two nutrition stages, including starter stage (1 to 21 d) and grower stage (22 to 42 d), were designed for this experiment. The average daily gain (ADG) and average daily feed intake (ADFI) of broilers at 1 to 21 d, 22 to 42 d, and 1 to 42 d were measured, and the feed conversion ratio (FCR) was calculated. The number of deaths of broilers was recorded and weighed daily, which was used to correct the FCR of broilers.

### Blood sample collection and analysis

On the 42nd day of age, two broilers with similar weights and fasted for 12 h were selected in each replicate for blood collection from the brachial wing vein. Approximately 5 mL was placed in an ethylenediaminetetraacetic acid dipotassium salt (EDTA-K2) tube and then stored at −20°C for hematological analysis. The white blood cell count (WBC), lymphocyte absolute value (LYM), intermediate cell absolute value (MID), granulocyte absolute value (GRA), red blood cell count (RBC), hemoglobin (Hb), hematocrit (HCT), platelet count (PLT), and platelet count (PCT) in the blood were determined by an automatic hematology analyzer (TEK8550, Jiangxi Tecom Technology Co., Ltd., Jiangxi, China).

### Serum sample collection and analysis

The blood samples were centrifuged at 3,000×g and 4°C for 15 min to collect serum, and the serum was stored at −80°C for future analysis. The contents of total protein (TP), albumin (ALB), globulin (GLB), alkaline phosphatase (ALP), alanine aminotransferase (ALT), lactate dehydrogenase (LDH), aspartate aminotransferase (AST), lipoprotein (LP), triglyceride (TG), total cholesterol (T-CHO), blood urea nitrogen (BUN) in serum were determined by the automatic biochemical analyzer (AU5800; American Beckman Coulter Co., Ltd., USA).

Serum antioxidant enzymes, total antioxidant capacity (T-AOC; A015-1-2, Colorimetric method), superoxide dismutase (SOD; A001-3-2, WST-1 method), glutathione peroxidase (GSH-Px; A005-1-2, Colorimetric method), malondialdehyde (MDA; A003-1-1, TBA method), immunoglobulin A (IgA, H108-1-2), immunoglobulin M (IgM, H109-1-2), and immunoglobulin G (IgG, H106-1-1) were measured according to the methods of assay kit. All experimental steps were carried out in strict accordance with the instructions of the reagent company (Nanjing Jiancheng Biotechnology Co., Ltd., Jiangsu, China).

### Statistical analysis

The experiment data were analyzed using one-way analysis of variance and the general linear model in SAS 9.4 (SAS Institute Inc., Cary, NC, USA). Variations among the treatments were compared using Tukey’s multiple range tests. Results were presented as the mean and standard error of the mean. All statements of significance are based on a probability of p<0.05.

## RESULTS

### Growth performance

Table 5 presents the impact of replacing SBM with CAP in broiler chicken diets on growth performance. In the start phase (1 to 21 d), there were no significant changes in ADG, ADFI, and FCR among the CON, CAP-1, and CAP-2 groups (p>0.05). In the grower phase (22 to 42 d), ADG was significantly increased in the CAP-1 group compared to the CON group (p<0.05), but not significantly different to the CAP-2 group (p>0.05). CAP-2 group showed lower ADFI compared to the CON and CAP-1 groups (p<0.05), with the CAP-1 group being intermediate (p<0.05). The FCR of the CAP-1 and CAP-2 groups were lower than the CON group, but there was no significantly variation between the treatment groups (p>0.05). Throughout the entirety of the experiment, spanning from 1 to 42 days, the CAP-1 and CAP-2 groups demonstrated higher ADG and lower FCR when compared to the CON group (p<0.05), but there was no significant difference observed between the CAP-1 and CAP-2 groups (p>0.05). Additionally, as the proportion of SBM in CAP substitute feed increased, the ADFI of broiler chickens in the CON, CAP-1, and CAP-2 groups decreased in a sequential manner (p<0.05).

### Blood routine

Table 6 displays the effects of the CAP substitute for SBM on the hematology of broiler chickens. The Hb in the blood of both the CAP-1 and CAP-2 groups showed a significant increase when compared to the CON group (p<0.05). However, there was no significant difference between the two treatment groups (p>0.05). Additionally, the HCT of the CAP-2 group was higher than the CON group (p<0.05), but there was no significant alteration to the CAP-1 group (p>0.05). The substitution of CAP for 25% and 50% SBM in the diet did not have a significant effect on blood WBC, LYM, MID, GRA, RBC, PLT, and PCT indexes (p>0.05).

### Serum biochemistry

Table 7 presents the impact of replacing SBM with CAP on the serum biochemical indicators in broilers. No statistically significantly differences were observed for the TP, ALB, ALP, GLB, LDH, ALT, AST, LP, TG, total cholesterol, and BUN in the CON, CAP-1, and CAP-2 groups (p>0.05).

### Serum antioxidant capacity

Table 8 shows the impact of CAP substitution for SBM on the serum antioxidant capacity of broiler chickens. The SOD activity of the CAP-2 group was increased compared to the CON and CAP-1 groups (p<0.05). Additionally, the levels of MDA content in the serum of both the CAP-1 and CAP-2 groups were significantly lower compared to the CON group (p<0.05). However, there was no significant difference observed between the CAP-1 and CAP-2 groups (p>0.05).

### Serum immunoglobulin content

The effect of CAP replacing SBM on the serum immunoglobulin content of broiler chickens is shown in Table 9. The study found that there was no significant difference in serum IgA levels between the CON and CAP-1 groups (p>0.05), but lower than the CAP-2 group (p<0.05). Compared to the CON group, the CAP-1 and CAP-2 groups in serum IgG content were significantly increased (p<0.05), and the CAP-2 group higher than the CAP-1 group (p<0.05). However, there was no significant alteration in serum IgM content between the CON group and treatment groups (p>0.05).

## DISCUSSION

Soybean meal is the main source of protein for China’s feed industry, which mostly depends on imports from abroad and is greatly affected by prices [15]. In recent years, the price of SBM continues to rise, and the demand exceeds supply [16]. The search for a new, efficient, and sustainable protein source to replace SBM is crucial to address the shortage of feed protein resources, while use of CAP can be a significant solution to this issue. The present study systematically analyzed the chemical composition, amino acid composition and ileal digestibility of CAP in Arbor Acres broiler chickens. The protein content of CAP (84.77%) was significantly higher than that of SBM (43% or 46%), and the content of amino acids, such as Met (2.29%), Lys (7.74%), and Thr (3.69%), were also significantly increased, which is more in line with the requirements of broilers for amino acids [17]. Additionally, the ileal apparent protein digestibility (82.04%) and standardized protein digestibility (93.81%) indicated that the high content of protein in CAP was easily digested and absorbed by broilers. Amino acids are an essential part of protein, and the amino acids required for growth and development of broiler chickens are mainly provided by the feed [18,19]. Most of the amino acids in the terminal ileum are digested and absorbed in the small intestine, which can truly reflect the digestion and absorption of amino acids in animals [20]. This experiment showed that the ileal digestibility of Lys, Met, Arg, Ile, Leu, Thr, Val, Ala, Gly, Glu, Pro, Ser, Cys, and Asp in broiler CAP were significantly higher than that of SBM, while the digestibility of His was slightly lower than that of SBM [2]. In feed formulation, Lys serves as the reference amino acid for the ideal protein model and is considered the most crucial amino acid for broiler chickens [21]. Met is the first limiting amino acid for broiler chickens, and it is of great significance in improving the growth performance, feed efficiency, and survival rate of broilers [22]. Furthermore, dietary amino acids such as limiting amino acids (Lys, Met, Arg, Thr, His, Try, Val, Leu, Iso, and Phe) and non-limiting amino acids (Gly, Ser, Pro, Ala, Asp, Glu, Cys, and Tyr) are essential for health [23–25]. Importantly, the experiment observed that the apparent metabolizable energy (4.2 kcal/g) and net energy (2.2 kcal/g) of CAP were superior to that of SBM [26]. Broiler metabolizable energy and net energy are important indicators for evaluating feed energy utilization efficiency [27–29]. In summary, substituting CAP for SBM can improve the utilization rate of energy and amino acids in broilers, which confirms the feasibility of using CAP in broilers.

Several researchers have investigated the use of CAP as an effective protein source for fish [30]. For example, replacing fish meal with CAP in the diet can improve the performance of farmed species [31]. However, few studies have been conducted on CAP in poultry. Previous studies found that the CAP instead of 8% to 36% SBM significantly increased the ADFI of Cobb broilers, and decreased F/G in the early growth period (1 to 21 d) and reduced the F/G in overall study [7]. The study found that replacing 25% and 50% SBM with CAP had no significant impact on the growth performance of broilers aged 1 to 21 days. However, in the later growth period, broilers supplemented with CAP showed increased ADG and decreased ADFI and FCR. Throughout the entire growth period, broilers supplemented with CAP had significantly increased ADG and decreased ADFI and FCR compared to the CON group. The production performance in this experiment differed from previous ones due to a higher proportion of CAP replacing SBM. It is worth noting that the deviation of test results can also be caused by the difference between broiler breeds or the CAP additive amount. Meanwhile, the small intestine is not fully developed at the early stage of growth, which leads to low digestion and absorption capacity of nutrients, and ultimately affected the improvement of broiler growth performance [32–34]. In comparison to SBM, CAP contains abundant amino acids that are easily absorbed by broilers, leading to positive growth performance in the late growth stage (22–42). Current production data suggests that increasing the amount of CAP in feed instead of SBM can further improve the performance of broiler chickens. As a result, the application of CAP in broiler chickens has significant potential for development.

Blood biochemical indicators are usually used to reflect changes in metabolism and organ function [35]. In this study, the replacement of SBM with 25% and 50% CAP significantly increased blood Hb and HCT levels in broilers. Hb and HCT are generally positively correlated with growth performance, antioxidant capacity, and immunity [36,37], which may be one of the reasons for improved broiler health. In contrast, the study found that the use of CAP-1 and CAP-2 did not have a significant impact on the serum biochemical indicators in broilers. This suggests that substituting SBM with 25% and 50% CAP as a protein supplement for broilers does not have any negative effects on their blood.

Broiler chickens are highly susceptible to feed, heat stress, and pathogen infection, resulting in a stress response in the early development of poultry [38,39]. Oxidative stress refers to the disruption of the balance between antioxidants and free radicals that generate various reactive oxygen species (ROS) [40,41]. ROS damages proteins and nucleic acids while producing a large amount of MDA, causing tissue damage, and reducing antioxidant capacity [42]. Antioxidant enzymes such as SOD and GSH-Px can remove excess ROS and maintain homeostasis in the internal environment [43,44]. In this study, the CAP-2 group significantly increased the SOD content in broiler serum. Serum MDA levels were significantly reduced in the CAP-1 and CAP-2 groups compared with CON group. This result is the same as previous studies [7], which showed that replacing SBM with 25% and 50% CAP significantly improved the antioxidant capacity of broilers, thereby reducing the damage caused by oxidative stress.

Previous studies have shown that increasing the antioxidant capacity of broiler chickens improves immune function [45]. IgA, IgM, and IgG are important immunoglobulins in broilers, which can resist viruses and toxins, protect the immune system, and maintain the health of the body [46,47]. IgG is secreted by plasma cells and has antiviral and antibacterial effects [40]. IgM has a strong bactericidal effect, activates antibodies, and regulates immune function [48]. As the first line of defense against pathogen invasion, IgA can slow down viral replication and improve intestinal barrier function [49]. Replacing SBM with 50% CAP significantly increased the serum IgA and IgG levels of broiler chickens, which may be enhance the immune function of broiler chickens by improving the intestinal mucosal barrier.

## CONCLUSION

This experiment systematically analyzed the chemical composition, amino acid composition, and ileal digestibility of CAP, and confirmed the feasibility of using high-level CAP instead of SBM in Arbor Acres broilers. The results showed that the protein content and amino acid composition of CAP were more conducive to the growth and development of Arbor Acres broilers. Moreover, the present experiment showed that CAP could increase the ADG of broilers, reduce the ADFI and FCR. Furthermore, the CAP replacement of SBM significantly improved the antioxidant capacity and immune function in broilers. In summary, the CAP has shown a positive role as a new protein material to replace SBM for broiler chickens, which provides an application of a new direction for non-grain feed sources.

## Figures and Tables

**Table 1:** The chemical composition, and amino acid composition of *Clostridium autoethanogenum* protein

Items^1)^	CAP	Soybean meal
Chemical composition (%, n = 6)		
DM	97.52	89.00
CP	84.77	46.00
EE	0.53	1.90
CF	4.53	5.90
Ash	5.80	6.10
Indispensable amino acids (%, n = 6)		
Lys	7.74	2.87
Met	2.29	0.61
Arg	2.63	3.23
Thr	3.69	1.70
His	1.87	1.18
Try	0.52	0.57
Val	4.98	2.24
Leu	5.52	3.49
Iso	4.96	1.82
Phe	3.06	2.21
Total IAA	37.36	19.92
Dispensable amino acids (%, n = 6)		
Gly	3.82	1.84
Ser	3.54	1.78
Pro	2.8	2.37
Ala	9.39	1.96
Asp	8.31	5.03
Glu	8.96	8.11
Cys	0.70	0.64
Tyr	0.52	1.60
Total DAA	38.04	23.33
Energy values (kcal/g, n = 6)		
AME	4.20	2.49
NE	2.20	1.58

CAP, *Clostridium autoethanogenum* protein; DM, dry matter; CP, crude protein; EE, ether extract; CF, crude fibre; Lys, lysine; Met, methionine; Arg, arginine; Thr, threonine; His, histidine; Try, tryptophan; Val, valine; Leu, leucine; Iso, isoleucine; Phe, phenylalanine; IAA, indispensable amino acids; Gly, glycine; Ser, serine; Pro, Proline; Ala, alanine; Asp, aspartic acid; Glu, glutamic; Cys, cystine; Tyr, tyrosine; DAA, dispensable amino acids; TIAA, total Indispensable amino acid; TDAA, total dispensable amino acid; AME, apparent metabolizable energy; NE, net energy.

**Table 2:** The dry matter and protein digestibility of C*lostridium autoethanogenum* protein in the broilers’ ileum

Items	Digestibility
Apparent ileal digestibility of dry matter (n = 6)	
Dry matter (%)	88.69
Apparent ileal digestibility of protein (n = 6)	
Crude protein (%)	82.04
Standardized ileal digestibility of protein (n = 6)	
Crude protein (%)	93.81

**Table 3:** The apparent and standardized amino acid digestibility of *Clostridium autoethanogenum* protein in the broilers’ ileum (n = 6)

Amino acids	Ileal amino acid digestibility	

	Apparent (%)	Standardized (%)
Lysine	94.77	94.73
Methionine	92.12	92.24
Arginine	91.18	91.41
Histidine	78.11	78.42
Isoleucine	90.37	90.42
Leucine	90.53	90.54
Threonine	85.69	85.96
Valine	89.80	89.86
Alanine	89.32	89.24
Glycine	86.39	86.74
Glutamic acid	89.26	89.10
Proline	85.26	85.52
Serine	84.47	84.79
Cysteine	72.43	73.39
Aspartic acid	88.63	88.38

**Table 4:** Ingredients and nutrient content of experimental diets (%, as-is basis)

Items	Contents					

	1 to 21 d			22 to 42 d		
	
	CON^1)^	CAP-1^1)^	CAP-2^1)^	CON^1)^	CAP-1^1)^	CAP-2^1)^
Corn	55.18	59.36	62.48	58.02	61.77	65.60
46% Soybean meal	30.26	24.92	19.69	23.98	19.02	13.95
84.77% CAP	0.00	2.50	5.00	0.00	2.50	5.00
Vegetable oil	2.06	0.86	0.00	3.08	1.94	0.76
49.42% Peanut meal	3.00	3.00	3.00	3.00	3.00	3.00
DDGS	5.00	5.00	5.00	8.00	8.00	8.00
NaCl	0.32	0.32	0.32	0.32	0.32	0.32
CaHPO_4_	1.73	1.75	1.79	1.44	1.47	1.50
Limestone	1.41	1.44	2.02	1.20	1.23	1.26
*L*-Lysine	0.39	0.30	0.21	0.37	0.28	0.19
*DL*-Methionine	0.32	0.21	0.13	0.28	0.17	0.11
*L*-Threonine	0.00	0.01	0.01	0.02	0.02	0.01
*L*-Tryptophan	0.00	0.00	0.02	0.00	0.00	0.02
Choline chloride	0.20	0.20	0.20	0.15	0.15	0.15
Premix^2)^	0.13	0.13	0.13	0.13	0.13	0.13
Nutrients^3)^						
Digestive energy (kcal/kg)	3,000	3,000	3,000	3,100	3,100	3,100
Crude protein	20.50	20.50	20.50	18.50	18.50	18.50
Calcium (%)	1.000	1.000	1.198	0.850	0.850	0.850
Phosphorus	0.679	0.660	0.641	0.624	0.607	0.589
Nonphytate phosphorus	0.450	0.450	0.450	0.420	0.420	0.420
Lysine (%)	1.250	1.250	1.250	1.100	1.100	1.100
Methionine (%)	0.632	0.553	0.500	0.566	0.490	0.450
Threonine (%)	0.800	0.800	0.803	0.720	0.720	0.720
Methionine+Cysteine (%)	0.950	0.950	0.975	0.850	0.850	0.885
Tryptophan (%)	0.260	0.240	0.240	0.221	0.201	0.200

CAP, *Clostridium autoethanogenum* protein; DDGS, dry distillers grains with solubles.

1) CON group, basal diet in control group; CAP-1 group, *Clostridium autoethanogenum* protein replaces 25% soybean meal in the diets; CAP-2 group, *Clostridium autoethanogenum* protein replaces 50% soybean meal in the diets.

2) The premix provided the following per kilogram diet: (1 to 21 d) vitamin A 10,000 IU, vitamin D_3_ 2,000 IU, vitamin E 10 IU, vitamin K_3_ 2.5 mg, vitamin B_1_ 1.8 mg, vitamin B_2_ 4 mg, vitamin B_3_ 50 mg, vitamin B_5_ 11 mg, vitamin B_9_ 0.5 mg, vitamin B_12_ 0.7 mg, biotin 0.12 mg, Cu (as copper sulfate) 8 mg, Fe (as ferrous sulfate) 80 mg, Mn (as manganese sulfate) 60 mg, Zn (as zinc sulfate) 80 mg, Se (as sodium selenite) 0.15 mg, I (as potassium iodide) 0.35 mg. (22 to 42 d) vitamin A 8,000 IU, vitamin D_3_ 1,500 IU, vitamin E 8 IU, vitamin K_3_ 2.0 mg, vitamin B_1_ 1.5 mg, vitamin B_2_ 3 mg, vitamin B_3_ 40 mg, vitamin B_5_ 9 mg, vitamin B_9_ 0.4 mg, vitamin B_12_ 0.6 mg, biotin 0.10 mg, Cu (as copper sulfate) 6 mg, Fe (as ferrous sulfate) 60 mg, Mn (as manganese sulfate) 50 mg, Zn (as zinc sulfate) 60 mg, Se (as sodium selenite) 0.12 mg, I (as potassium iodide) 0.30 mg.

3) Digestive energy, amino acids, calcium, phosphorus, and nonphytate phosphorus are calculated values, while other values are measured values.

**Table 5:** Effects of *Clostridium autoethanogenum* protein substitute for soybean meal on growth performance of broiler chickens

Items	Group^1)^			SEM	p-value

	CON	CAP-1	CAP-2		
1 to 21 d					
ADG (g)	31.99	30.58	31.02	0.648	0.543
ADFI (g/d)	46.89	45.91	46.87	0.926	0.482
FCR	1.51	1.50	1.51	0.038	0.892
22 to 42 d					
ADG (g)	68.65^b^	75.04^a^	73.01^ab^	1.063	0.038
ADFI (g/d)	87.60^a^	84.53^b^	79.55^c^	3.391	<0.001
FCR	1.28^a^	1.13^b^	1.09^b^	0.046	0.035
1 to 42 d					
ADG (g)	50.32^b^	52.81^a^	52.02^a^	1.162	0.032
ADFI (g/d)	67.25^a^	65.22^b^	63.21^c^	1.611	<0.001
FCR	1.34^a^	1.23b	1.22^b^	0.021	0.021

SEM, standard error of the mean; ADG, average daily gain; ADFI, average daily feed intake; FCR, feed conversion rate.

1) CON group, basal diet in control group; CAP-1 group, *Clostridium autoethanogenum* protein replaces 25% soybean meal in the diets; CAP-2 group, *Clostridium autoethanogenum* protein replaces 50% soybean meal in the diets.

a–c Means followed by different letters in a row differ significantly (p<0.05).

**Table 6:** Effects of *Clostridium autoethanogenum* protein substitute for soybean meal on blood routine of broiler chickens

Items	Group^1)^			SEM	p-value

	CON	CAP-1	CAP-2		
WBC (10^9^/L)	111.22	115.07	113.3	1.258	0.485
LYM (10^9^/L)	63.57	61.13	61.8	0.452	0.065
MID (10^9^/L)	15.84	16.48	16.14	0.213	0.498
GRA (10^9^/L)	31.81	37.46	35.37	1.350	0.235
RBC (10^12^/L)	2.59	2.76	2.85	0.052	0.115
Hb (g/L)	95.33^b^	106.67^a^	109.83^a^	2.415	0.024
HCT (L/L)	0.235^b^	0.256^ab^	0.269^a^	0.006	0.029
PLT (10^9^/L)	14.17	16.5	9.33	1.538	0.153
PCT (L/L)	0.02	0.02	0.01	0.002	0.176

SEM, standard error of the mean; WBC, white blood cell count; LYM, lymphocyte absolute value; MID, intermediate cell absolute value; GRA, granulocyte absolute value; RBC, red blood cell count; Hb, hemoglobin; HCT, hematocrit; PLT, platelet count; PCT, platelet count.

1) CON group, basal diet in control group; CAP-1 group, *Clostridium autoethanogenum* protein replaces 25% soybean meal in the diets; CAP-2 group, *Clostridium autoethanogenum* protein replaces 50% soybean meal in the diets.

a,b Means followed by different letters in a row differ significantly (p<0.05).

**Table 7:** Effects of Clostridium autoethanogenum protein substitute for soybean meal on serum parameters of broiler chickens

Items	Group^1)^			SEM	p-value

	CON	CAP-1	CAP-2		
TP (mg/mL)	115.04	107.55	122.67	4.413	0.399
ALB (mg/mL)	36.63	35.44	39.12	1.715	0.697
ALP (ng/mL)	122.1	148.68	145.46	0.065	0.149
GLB (g/L)	21.25	22.83	25.66	1.158	0.308
LDH (ng/mL)	10.92	12.28	14.39	0.623	0.062
ALT (mmoL/L)	124.71	184.26	156.76	11.569	0.104
AST (ng/mL)	260.05	250.97	258.11	10.680	0.943
LP (μg/mL)	287.78	264.87	298.83	13.166	0.590
TG (mg/mL)	0.43	0.47	0.51	0.023	0.437
T-CHO (μmol/dL)	961.4	1,043.7	1,141.2	55.390	0.441
BUN (mg/mL)	938.8	1,019.2	1,114.4	54.094	0.441

SEM, standard error of the mean; TP, total protein; ALB, albumin; ALP, alkaline phosphatase; GLB, globulin; LDH, lactate dehydrogenase; ALT, alanine aminotransferase; AST, aspartate aminotransferase; LP, lipoprotein; TG, triglyceride; T-CHO, total cholesterol; BUN, blood urea nitrogen.

1) CON group, basal diet in control group; CAP-1 group, *Clostridium autoethanogenum* protein replaces 25% soybean meal in the diets; CAP-2 group, *Clostridium autoethanogenum* protein replaces 50% soybean meal in the diets.

**Table 8:** Effects of *Clostridium autoethanogenum* protein substitute for soybean meal in the diet on serum antioxidant capacity of broiler chickens

Items	Group^1)^			SEM	p-value

	CON	CAP-1	CAP-2		
T-AOC (U/mL)	0.17	0.18	0.19	0.007	0.441
SOD (U/mL)	218.94^b^	252.49^b^	331.94^a^	16.662	0.008
MDA (nmoL/mL)	12.28^a^	9.92^b^	9.98^b^	0.401	0.012
GSH-Px (U/mL)	147.01	135.47	135.48	4.128	0.446

n = 6.

SEM, standard error of the mean; T-AOC, total antioxidant capacity; SOD, superoxide dismutase; MDA, malondialdehyde; GSH-Px, glutathione peroxidase.

1) CON group, basal diet in control group; CAP-1 group, *Clostridium autoethanogenum* protein replaces 25% soybean meal in the diets; CAP-2 group, *Clostridium autoethanogenum* protein replaces 50% soybean meal in the diets.

a,b In the same row, values with different small letter superscripts mean significant difference (p<0.05).

**Table 9:** Effects of *Clostridium autoethanogenum* protein substitute for soybean meal in the diet on serum immunoglobulin content of broiler chickens

Items (μg/mL)	Group^1)^			SEM	p-value

	CON	CAP-1	CAP-2		
IgA	142.35^b^	165.59^b^	207.92^a^	10.686	0.007
IgM	614.95	629.39	640.34	9.859	0.103
IgG	1,328.03^c^	1,450.93^b^	1,769.05^a^	66.852	<0.001

n = 6.

SEM, standard error of the mean; IgA, immunoglobulin A; IgM, immunoglobulin M; IgG, immunoglobulin G.

1) CON group, basal diet in control group; CAP-1 group, *Clostridium autoethanogenum* protein replaces 25% soybean meal in the diets; CAP-2 group, *Clostridium autoethanogenum* protein replaces 50% soybean meal in the diets.

a–c In the same row, values with different small letter superscripts mean significant difference (p<0.05).

## Data Availability

The datasets analysed in the current study are available from the corresponding author on request.
